# Supplementary material for: Atomically dispersed MoNi alloy catalyst for partial oxidation of methane
Source: Nat Commun. 2024 May 31;15:4636. doi: 10.1038/s41467-024-49038-x (PMC11143339; doi:10.1038/s41467-024-49038-x)
Supplement: Supplementary file 1 — Supplementary Information [file 41467_2024_49038_MOESM1_ESM.pdf]

Supplementary Information

Atomically Dispersed MoNi Alloy Catalyst for Partial Oxidation of Methane

Zheyuan Ding[1,2#], Sai Chen[1,2#], Tingting Yang[1,2,3], Zunrong Sheng[1,2], Xianhua Zhang[1,2], Chunlei Pei[1,2], Donglong Fu[1,2], Zhi-Jian Zhao[1,2], and Jinlong Gong[1,2,3,4,5*]

[1]*Key Laboratory for Green Chemical Technology of Ministry of Education, School of Chemical Engineering & Technology, Collaborative Innovation Center for Chemical Science & Engineering, Tianjin University, Tianjin 300072, China*

[2]*Collaborative Innovation Center for Chemical Science & Engineering (Tianjin), Tianjin 300072, China*

[3]*Joint School of National University of Singapore and Tianjin University, International Campus of Tianjin University, Binhai New City, Fuzhou 350207, China*

[4]*Haihe Laboratory of Sustainable Chemical Transformations, Tianjin, China*

[5]*National Industry-Education Platform of Energy Storage, Tianjin University, Tianjin, China*

#These authors contributed equally to this work.

*Correspondence: jlgong@tju.edu.cn

This PDF file includes

1. Figs. S1 to S23
2. Tables S1 to S7
3. References

Supplementary figures and tables

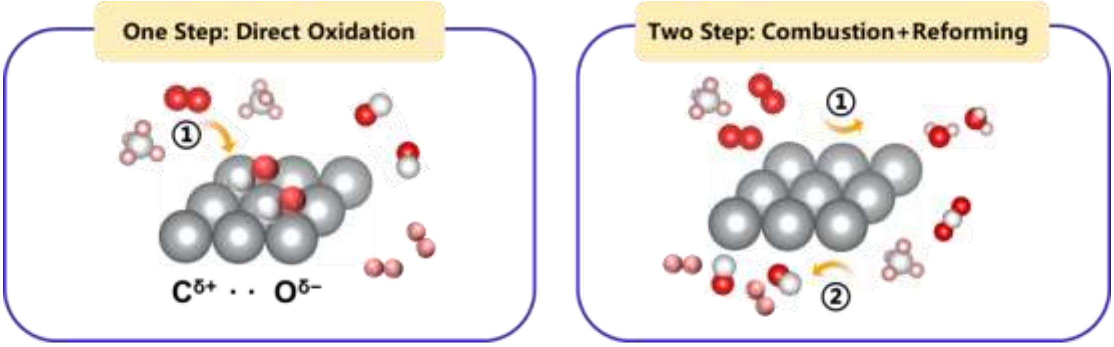

Figure S1 | Direct oxidation (Left) and two step combustion and reforming mechanism (Right) were proposed as main POM mechanism.

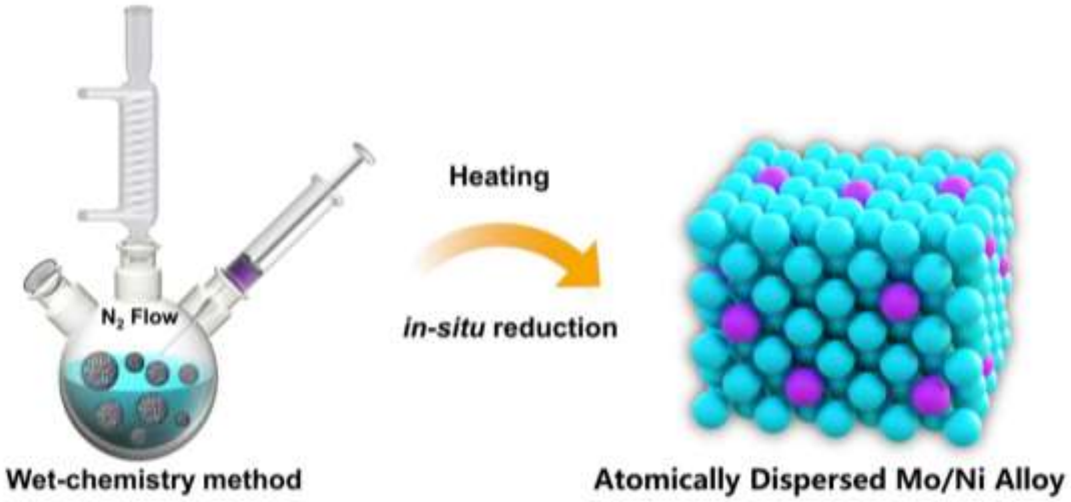

Figure S2 | Synthetic methods for alloy. Scheme of atomically dispersed MoNi alloy synthesis by wet-chemistry method under N_2 flow.

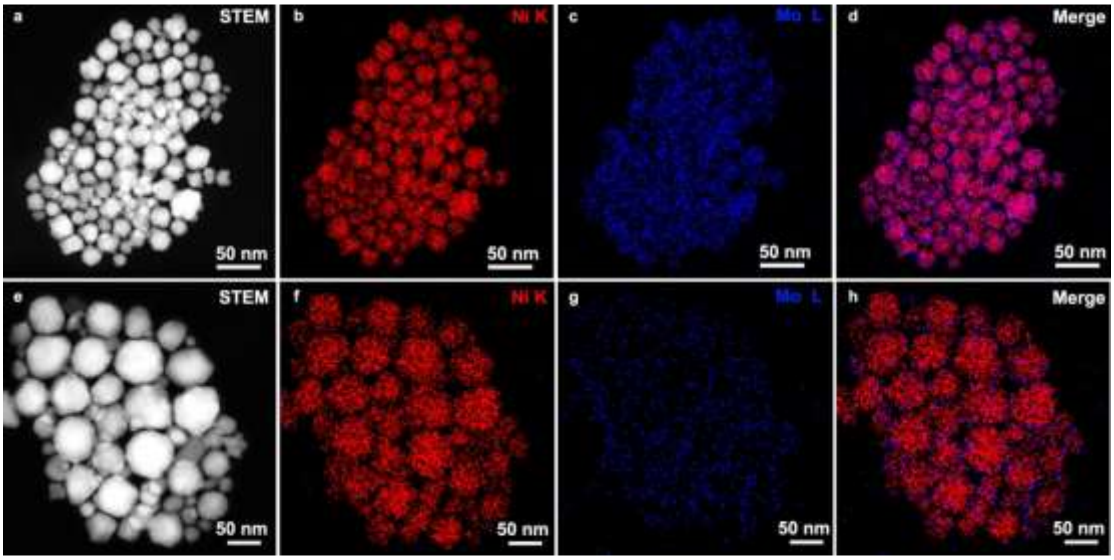

Figure S3 | Elemental distribution for as-prepared alloy. EDS elemental mapping of 5Ni1Mo alloy and AD MoNi alloy. (a) HAADF-STEM, (b) Ni K, (c) Mo L and (d) merge of 5Ni1Mo alloy. (e) HAADF-STEM, (f) Ni K, (g) Mo L and (h) merge of AD MoNi alloy.

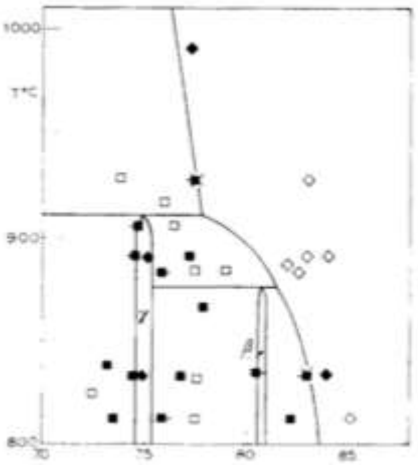

Figure S4 | Phase diagram for Mo-Ni alloys. Phase diagram of Ni-rich Mo-Ni alloys (70-90 at. % Ni), and *x* axis refer to atomic content and *y* axis stands for temperature. Adapted from R. E. W. Casselton and W. Hume-Rothery, *Journal of the Less-Common Metals.* 1964, 7, 212-221.

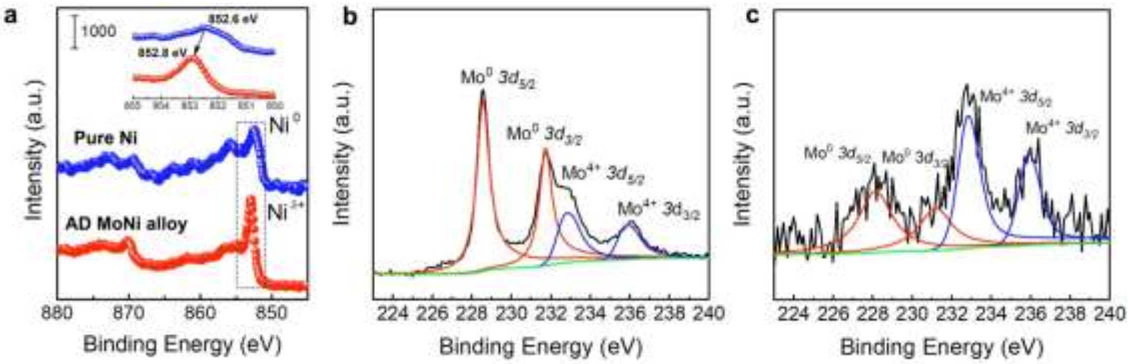

Figure S5 | Identification of valance state for fresh and spent catalysts. (a) XPS spectra of Ni 2*p* showing valence state variation for Ni/SBA-15 and AD MoNi alloy/SBA-15. Mo 3*d* spectra for fresh (b) and spent (c) AD MoNi alloy/SBA-15.

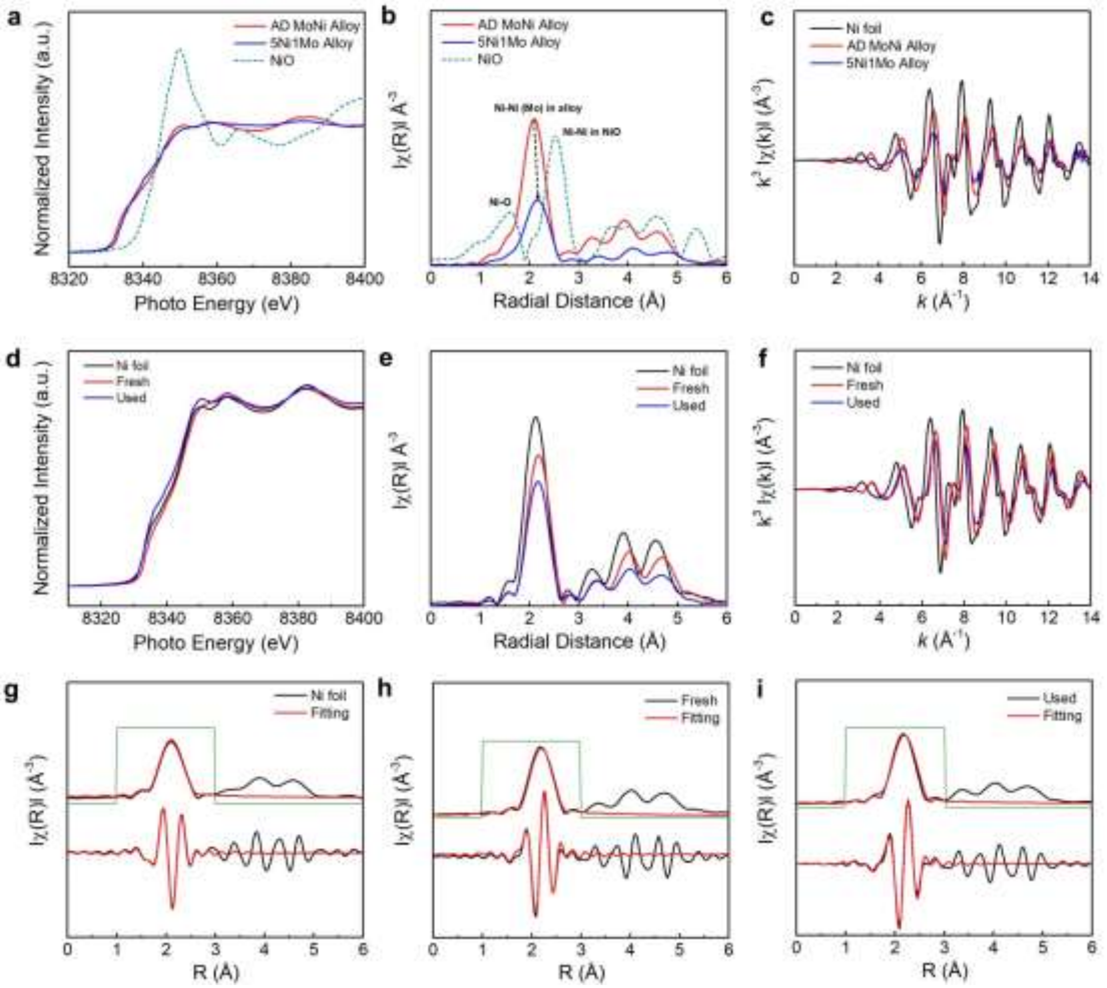

Figure S6 | XAFS analysis and fitting for electronic and coordination structure. (a) XANES spectra, (b) FT of k^3-weighted EXAFS R-space spectra and (c) *k* space for MoNi alloy and NiO reference. (d) XANES spectra, (e) FT of k^3-weighted EXAFS R-space spectra and (f) *k* space for fresh and spent AD MoNi alloy and Ni foil. FT of EXAFS R-space fitting for (g) Ni foil, (h) fresh AD MoNi alloy and (i) used AD MoNi alloy.

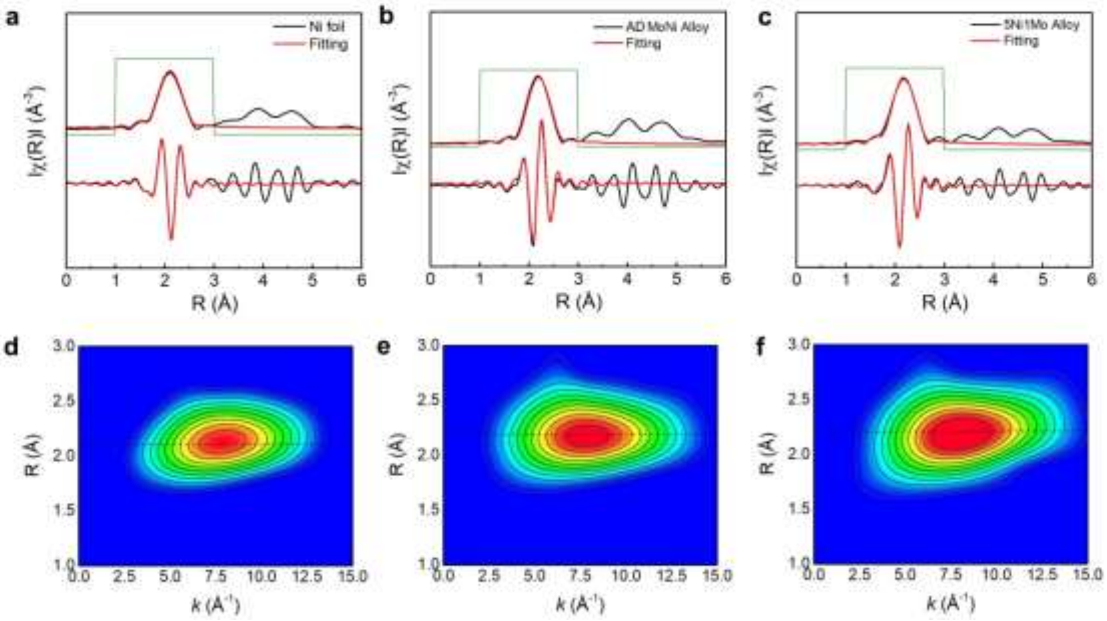

Figure S7 | XAFS fitting and treatment. EXAFS R-space fitting for (a) Ni foil, (b) AD MoNi alloy and (c) 5Ni1Mo alloy. Fitting window from 1.0 to 3.0 Å marked with green line. Wavelet transform (WT) of (d) Ni foil, (e) AD MoNi alloy and (f) 5Ni1Mo alloy, indicative of single peak feature.

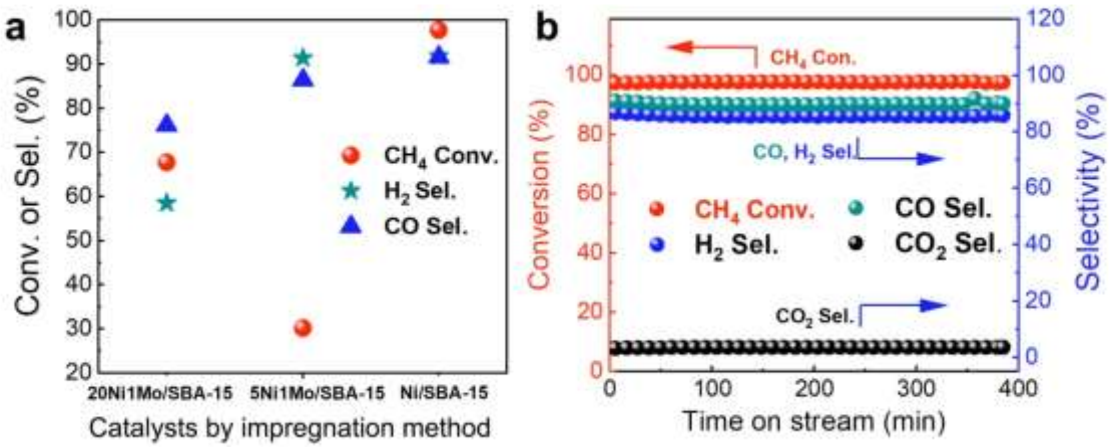

Figure S8 | Catalytic performance. (a) Performance test for 20Ni1Mo/SBA-15, 5Ni1Mo/SBA-15 and Ni/SBA-15 by impregnation synthetic method under 800 °C in a CH_4/Air (CH_4:O_2=2:1) flow with GHSV of 12,000 mL_{CH4} g_{cat}^{-1} h^{-1}. (b) Long-term stability test of Ni under 800 °C in a CH_4/Air (CH_4:O_2=2:1) flow with GHSV of 12,000 mL_{CH4} g_{cat}^{-1} h^{-1}.

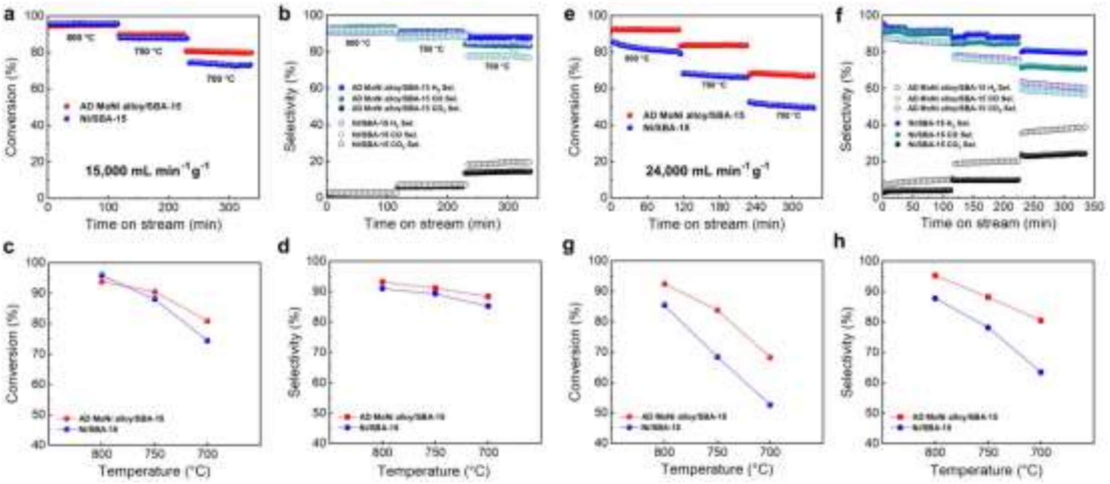

Figure S9 | Performance comparison between AD Mo/Ni alloy/SBA15 and Ni/SBA15 On-stream profiles of (a) Conversion and (b) Selectivity, and comparison of (c) Conversion and (d) Selectivity with GHSV of 15,000 mL_{CH4} g_{cat}^{-1} h^{-1} from 800 to 700 °C for AD Mo/Ni alloy/SBA15 and Ni/SBA15, respectively. On-stream profiles of (e) Conversion and (f) Selectivity, and comparison of (g) Conversion and (H) Selectivity with GHSV of 24,000 mL_{CH4} g_{cat}^{-1} h^{-1} from 800 to 700 °C for AD Mo/Ni alloy/SBA15 and Ni/SBA15, respectively.

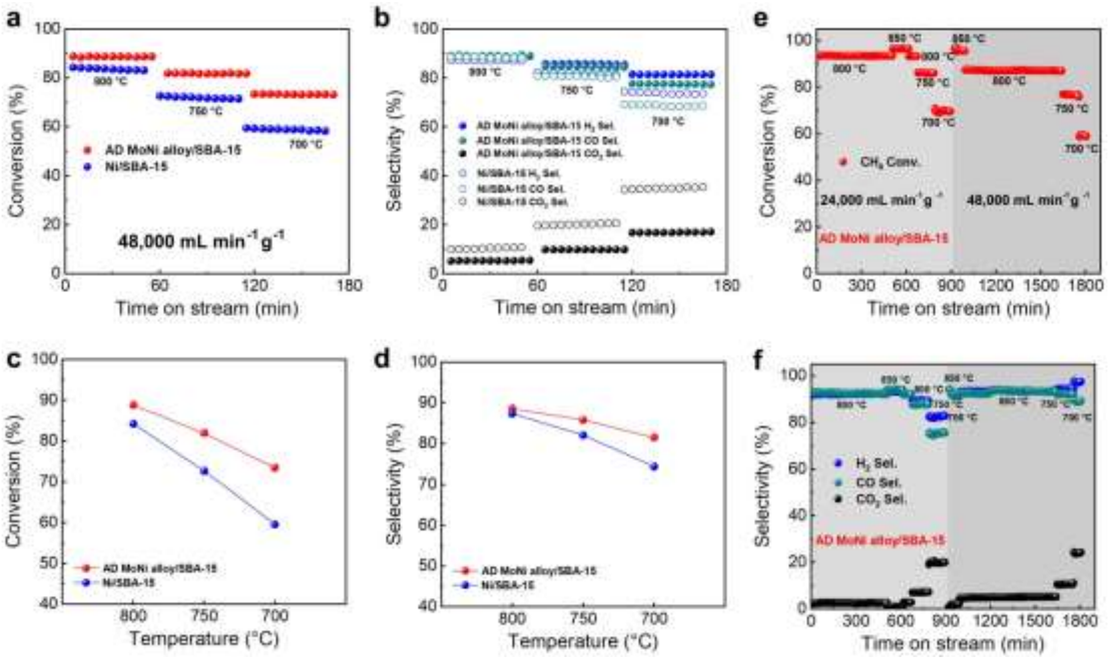

Figure S10 | Performance comparison between AD Mo/Ni alloy/SBA15 and Ni/SBA15. On-stream profiles of (a) Conversion and (b) Selectivity, and comparison of (c) Conversion and (d) Selectivity with GHSV of 48,000 mL_{CH4} g_{cat}^{-1} h^{-1}. from 800 to 700 °C for AD Mo/Ni alloy/SBA15 and Ni/SBA15, respectively. On-stream profiles of (e) Conversion and (f) Selectivity of AD Mo/Ni alloy/SBA15 with temperature switched from 850 to 700 °C and GHSV from 24,000 to 48,000 mL_{CH4} g_{cat}^{-1} h^{-1}.

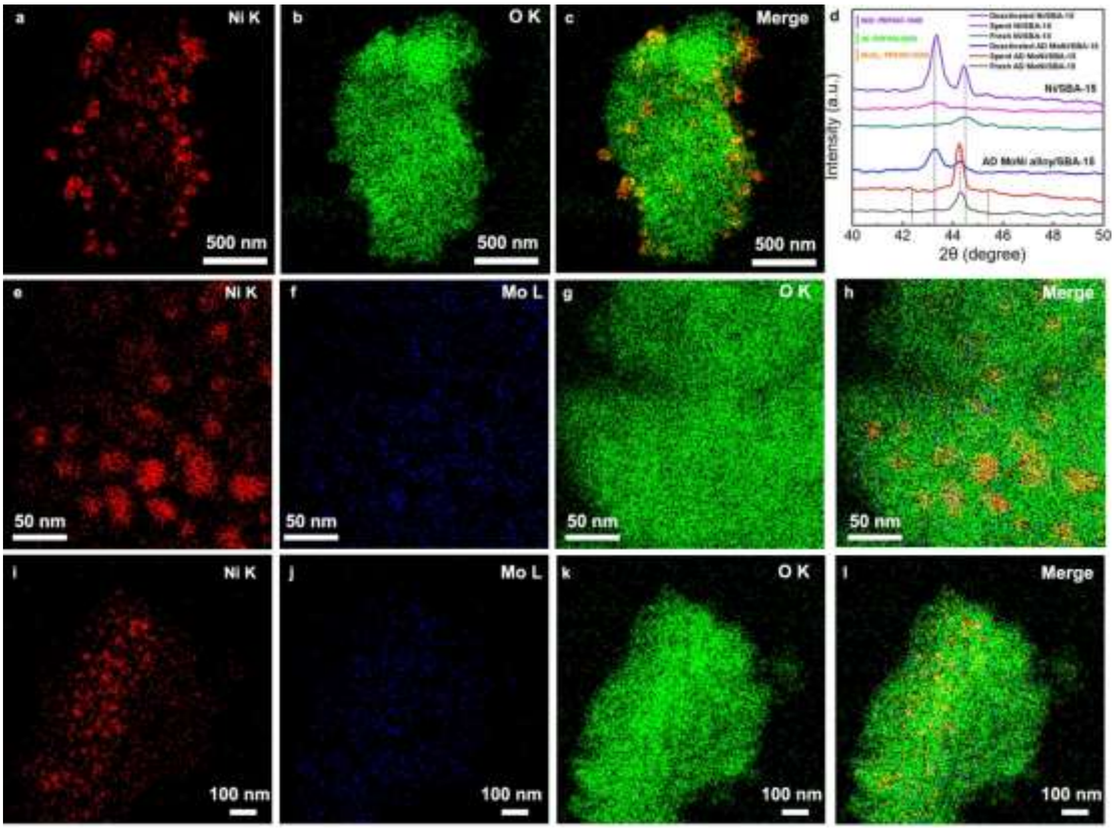

Figure S11 | Elemental distribution for fresh and spent catalyst. EDS mapping for (a) Ni K, (b) O K, (c) merge of pure Ni/SBA-15 catalysts (d) XRD for deactivated, spent (20h test) and fresh Ni/SBA-15 catalysts, and the deactivated, spent (20h test) and fresh AD MoNi alloy/SBA-15 catalysts. EDS mapping of (e) Ni K, (f) Mo L, (g) O K and (h) merge of AD MoNi alloy/SBA-15. EDS mapping of (i) Ni K, (j) Mo L, (k) O K and (l) merge of spent AD MoNi alloy/SBA-15 for POM reaction.

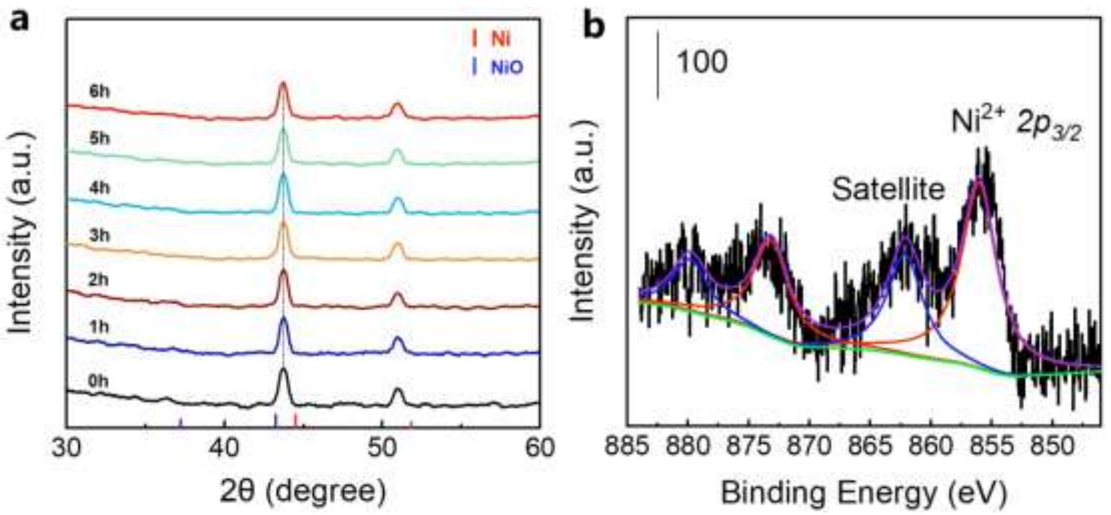

Figure S12 | *In-situ* experiments for active site identification. (a) The *in-situ* XRD test for AD MoNi alloy/SBA-15 catalyst under testing condition at 800 °C with CH_4/Air (CH_4:O_2=2:1) flow, indicative of alloy phase as active site for POM. (b) Quasi *in-situ* XPS of Ni/SBA-15 under reaction gas of CH_4/O_2/Ar (3:1.5:95.5) with total flow of 50 *sccm* at 800 °C.

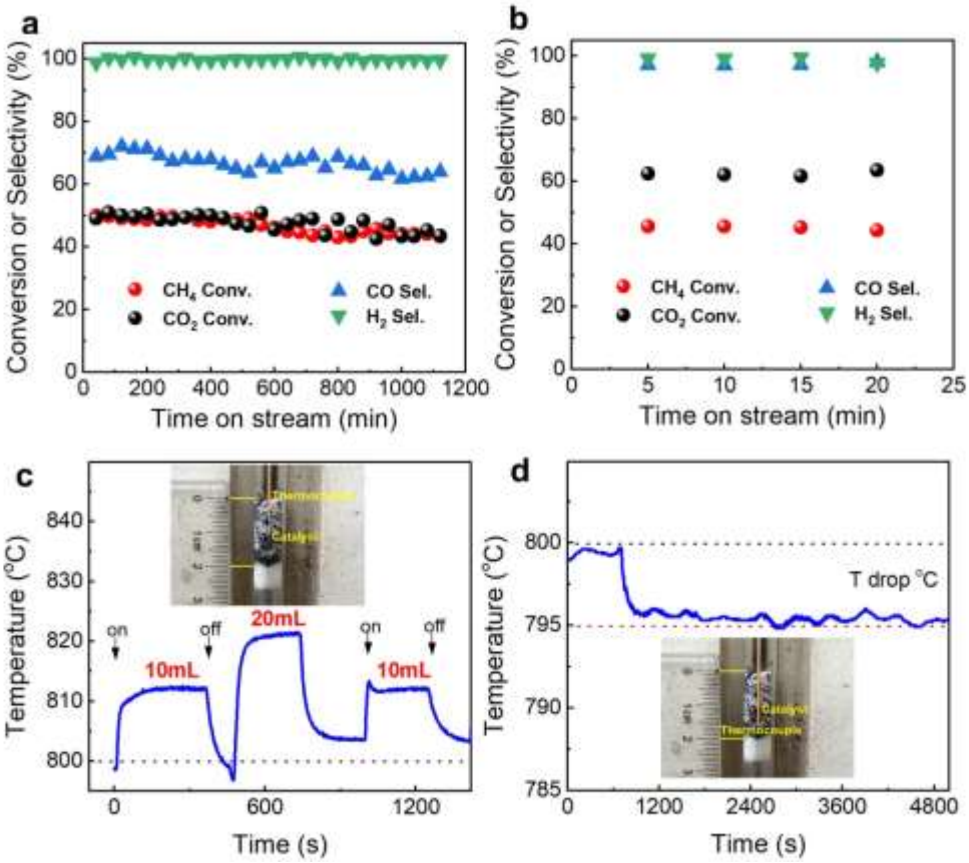

Figure S13 | Control experiments for identification of POM mechanism. Long-term test for (a) DRM and (b) DRM+SRM. (c) Temperature distribution located in gas entry bed (c) and gas exit bed (d) with obvious temperature drop. Scale bar is given within the digital image.

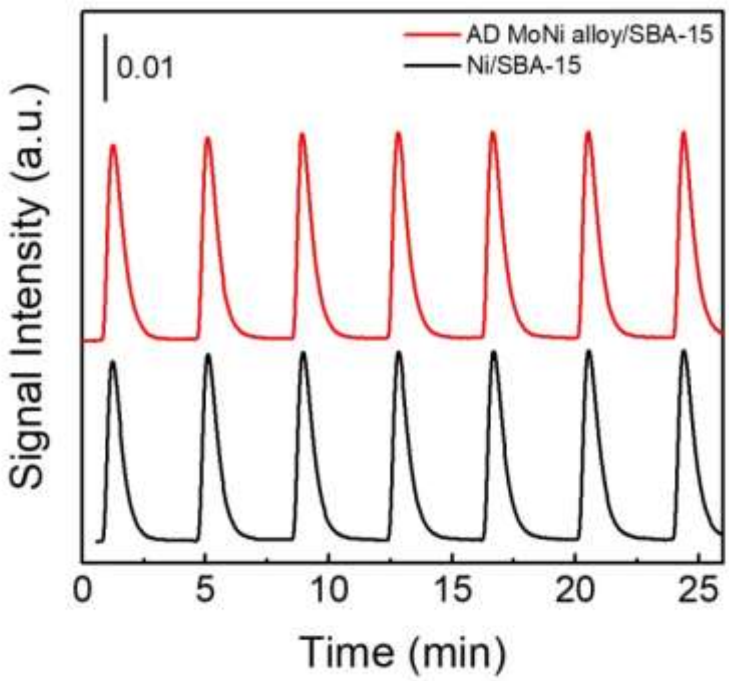

Figure S14 | Dispersion of catalysts. Dispersion of surface Ni atom for AD MoNi alloy/SBA-15 and Ni/SBA-15 by H_2-pulse chemisorption.

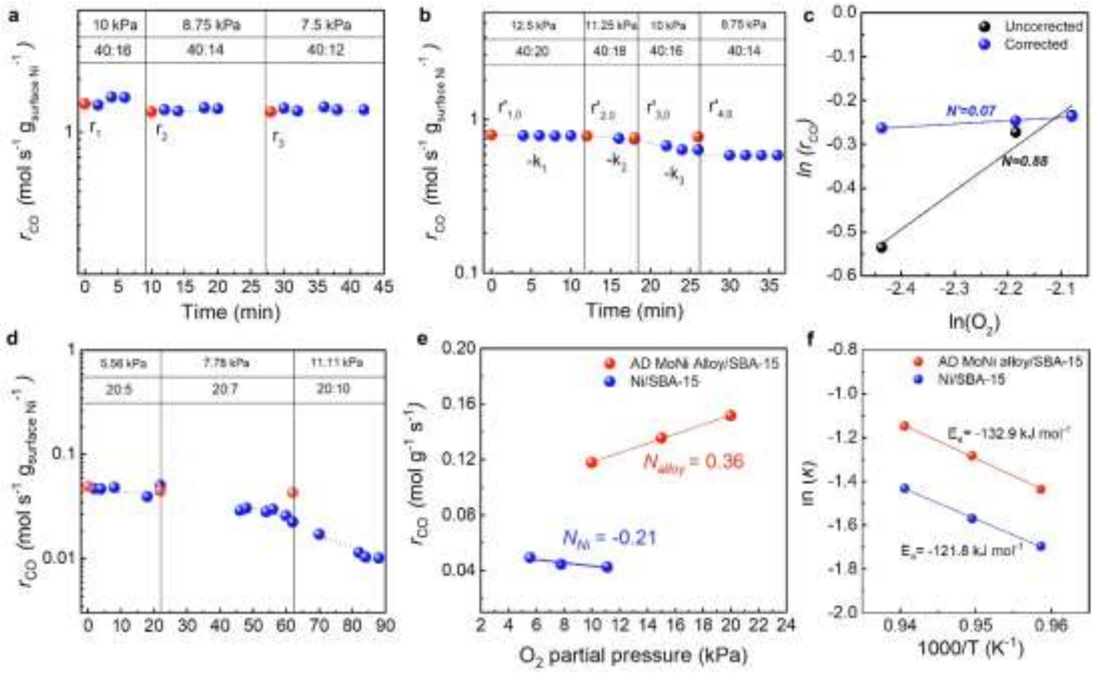

Figure S15 | Deactivation rate correction and kinetics tests. Runlog image for (a) AD MoNi alloy/SBA-15 and (b) Ni/SBA-15 to calculate the reaction order of O_2. Indicative of Ni/SBA-15 gradual deactivation and AD MoNi alloy/SBA-15 rarely deactivated when conducting kinetics study at 800 °C. The term r_i (i=1,2,3) in (a) is the real forward rate without correction due to the rarely deactivated behavior. The term $r'_{i,0}$ (i=1,2,3,4) in (b) is the corrected forward rate without deactivation at zero time. (c) Reaction order of O_2 for pure Ni/SBA-15 after correcting rate could be observed in Figure 4D. (d) Runlog for Ni/SBA-15 where red dots represents corrected forward rate without deactivation at zero time (e) Reaction order of O_2 at 670 °C (f) Arrhenius plot for AD MoNi alloy/SBA-15 and Ni/SBA-15 from 790 to 770 °C.

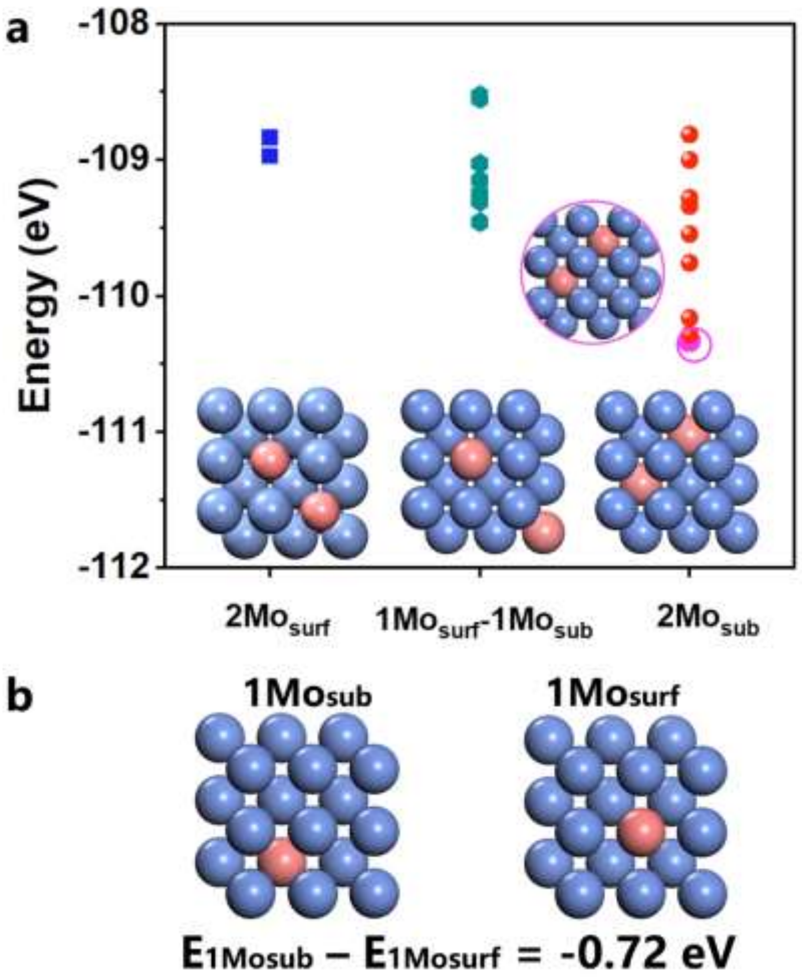

Figure S16 | Formation energy for possible structure determination. (a) Energies of surface models with 2 Mo atoms at different position. Inset represent corresponding schematic diagram (The structure marked by purple circle represents the most stable structure). Possible structure for AD MoNi alloy and its energy, where the two Mo atoms located at sub-surface for MoNi structure was the most stable structure with most negative energy. (b) Energy difference of -0.72 eV between $1Mo_{sub}$ and $1Mo_{surf}$ with a single Mo atom.

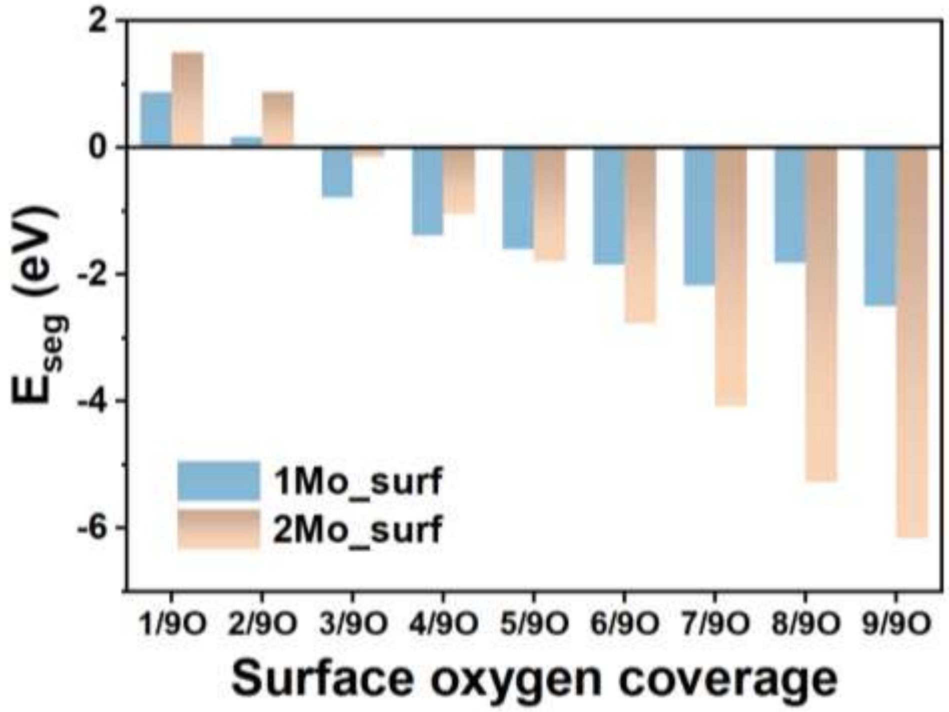

Figure S17 | Segregation energy for possible structure determination. Segregation energy of MoNi (100) with different amount of adsorbed O species.

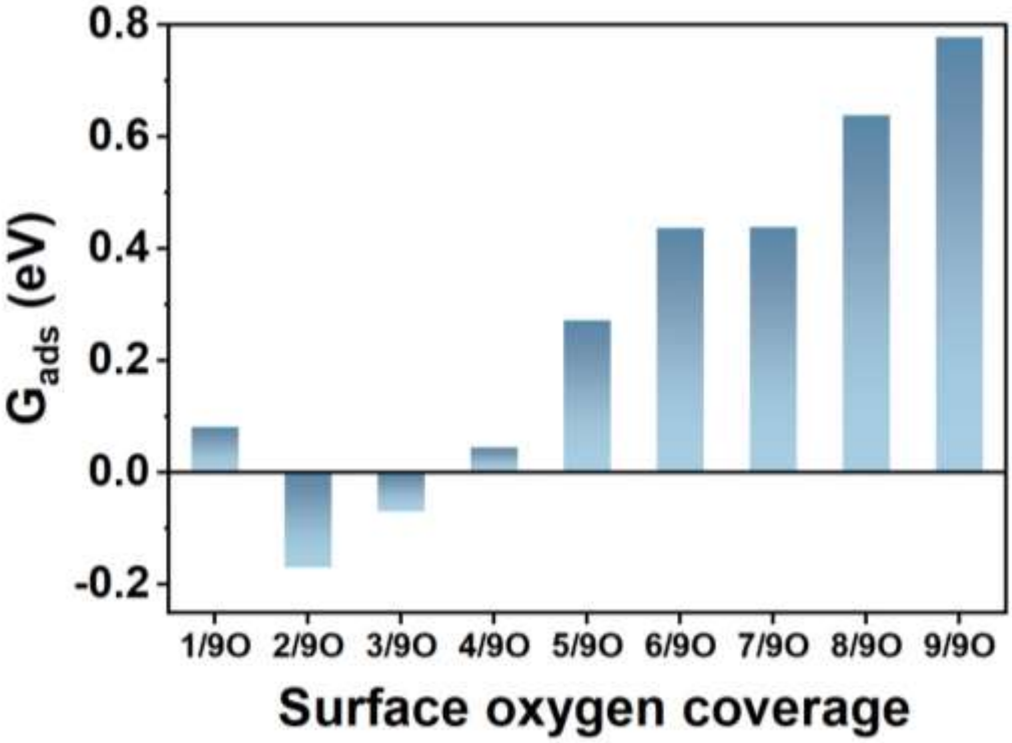

Figure S18 | Gibbs free adsorption energy. The variations of average oxygen Gibbs free adsorption energy on 1Mosurf-1Mosub surface with different surface oxygen coverage.

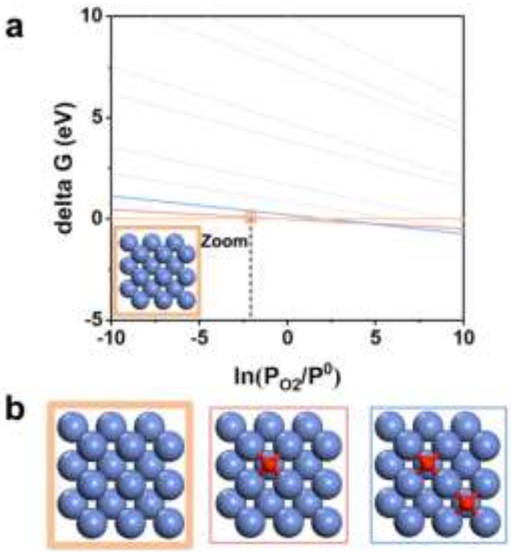

Figure S19 | Phase diagram for Ni. (a) Phase diagram for possible structure with practical O_2 partial pressure of ~0.125 under reaction condition at 800 °C with partial pressure of CH_4:O_2:N_2=2:1:5. (b) Possible structure according to phase diagram.

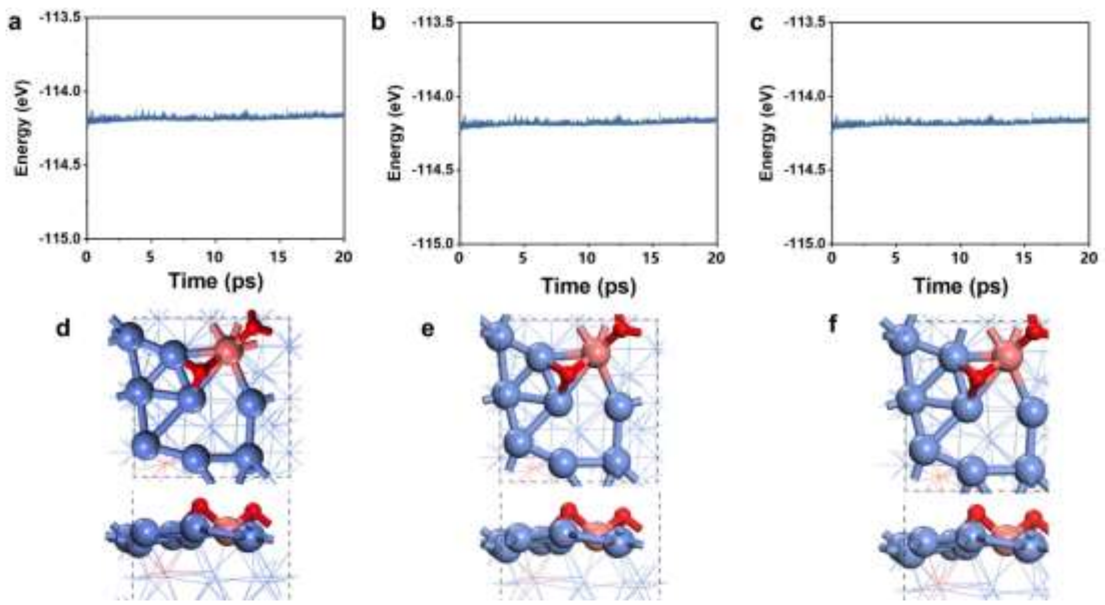

Figure S20 | Ab initio molecular dynamics (AIMD) for AD MoNi alloy. (a-c) Three repeated AIMD simulations were conducted at 1073 K within the NVT ensemble for 20ps. (d-f) The atomic structures represent MoNi (100) after simulation. Pre-adsorbed O atoms remain stable for 20 ps under reaction condition.

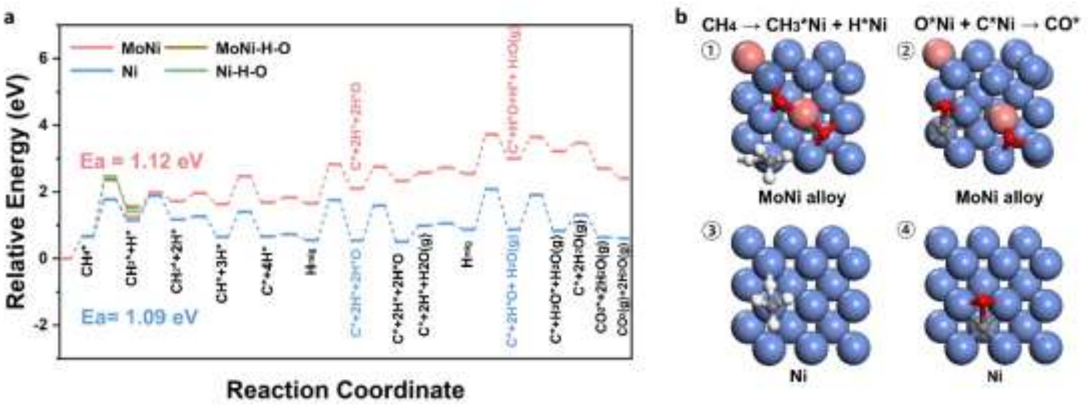

Figure S21 | Reaction pathways for MoNi (100) and Ni (100). Transition state of methane and oxygen on (a) MoNi (100) and (b) Ni (100).

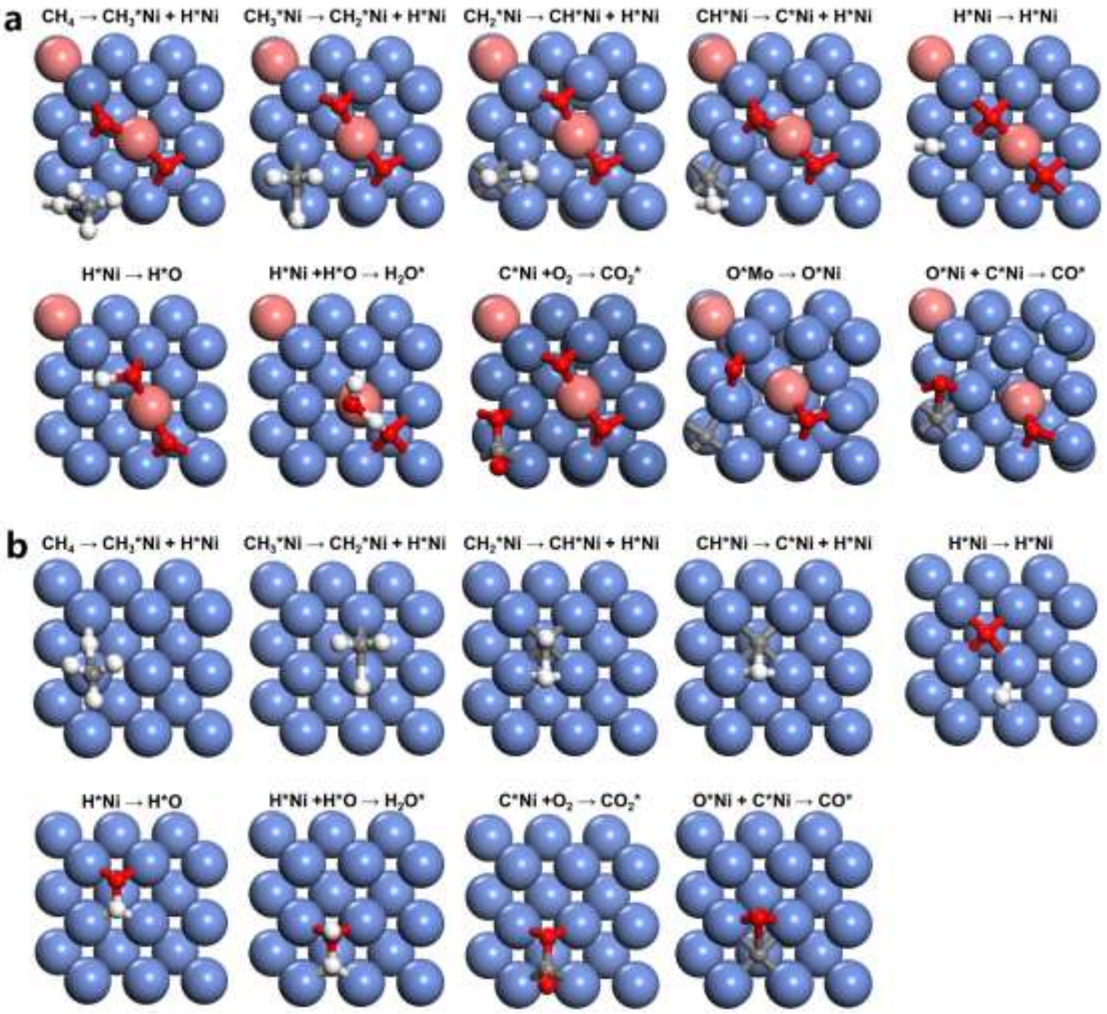

Figure S22 | Reaction pathways for MoNi (100) and Ni (100). Schematic structures for transition state of methane and oxygen on (a) MoNi (100) and (b) Ni (100).

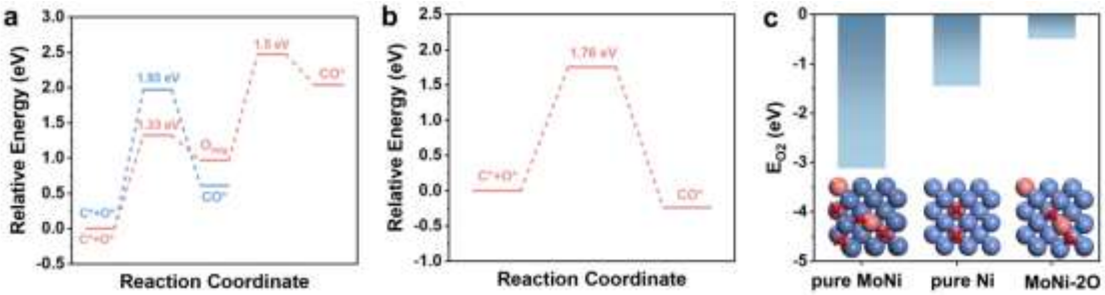

Figure S23 | Reaction pathways for MoNi (100) and Ni (100). Reaction pathways of C* with O* during methane reforming on (a) MoNi and (b) Ni. (c) The dissociative adsorption energy of O_2 on pure MoNi, pure Ni and MoNi-2O.

Table S1 EXAFS fitting parameters at the Ni K-edge

Sample	Shell	CN [a]	R (Å) [b]	σ^2(Å^2) [c]	R factor [d]	ΔE_0 (eV) [e]
**Ni foil**	Ni-Ni	12	2.48±0.01	0.007	0.007	-7.6
**AD Mo/Ni Alloy**	Ni-Ni	10.5±0.9	2.56±0.03	0.008	0.012	-0.4
**5Ni1Mo Alloy**	Ni-Ni	7.4±0.8	2.59±0.01	0.007	0.013	1.7

Note: [a] CN: coordination numbers; [b] R: bond distance; [c] σ^2 : Debye-Waller factors; [d] R factor: goodness of fit. [e] ΔE_0: the inner potential correction. S_0^2 was set as 0.98, which was obtained from the experimental EXAFS fit of NiO by fixing CN as the known crystallographic value and was fixed to all the samples.

Table S2 EXAFS fitting parameters at the Ni K-edge for reacted sample

Sample	Shell	CN [a]	R (Å) [b]	σ^2(Å^2) [c]	R factor [d]	ΔE_0 (eV) [e]
**Ni foil**	Ni-Ni	12	2.48±0.01	0.007	0.007	-7.6
**AD Mo/Ni Alloy used**	Ni-Ni	9.0±0.8	2.56±0.03	0.006	0.010	-0.9
**AD Mo/Ni Alloy fresh**	Ni-Ni	10.5±0.9	2.56±0.03	0.008	0.012	-0.4

Note: [a] CN: coordination numbers; [b] R: bond distance; [c]σ^2 : Debye-Waller factors; [d] R factor: goodness of fit. [e]ΔE_0: the inner potential correction. S_0^2 was set as 0.98, which was obtained from the experimental EXAFS fit of NiO by fixing CN as the known crystallographic value and was fixed to all the samples.

Table S3 Performance comparison of Ni based catalyst

**Cat.**	**Con. (%)** [a] **Sel. (%)** [b]	**T (°C)**	Notes	Ref.
Ni-CeO_2 /SiO_2	60% 60%	500 °C		[1]
Ni/CeO_2	92% 83%	650 °C		[2]
Ni/CeO_2 crystal	98% 83%	800 °C		[3]
Ni/CeO_2	~90% ~90%	650 °C		[4]
Ni/meso-SiO_2	93% -	750 °C		[5]
Ni-Co/SiO_2	84.1% 91.3%	700 °C		[6]
Ni/$CeZrO_2$	80% 90%	700 °C		[7]
Ni/$MgAl_2O_4$	89%	800 °C	Air as oxidants	[8]
Ni/CeO_2-La_2O_3	~80% ~90%	700 °C		[9]
Ni/MgO	75% 73%	800 °C		[10]
24.1 wt% Ni/Al_2O_3	98.5% 98.5%	850 °C	24.1 wt% loading	[11]
15 wt% NiO/CeO_2	~100% ~100%	800 °C	20% decrease for 20h	[12]
**AD Mo/Ni alloy/SBA15**	**~95% 97.7%**	**800 °C**	**Air as oxidants**	**This Work**

[a]CH_4 conversion, [b]CO selectivity

Table S4 Performance comparison of noble metal based catalyst

Cat.	Con. (%) [a] Sel. (%) [b]	T (°C)	Notes	Ref.
Rh sub-nano clusters/Zoelite	84% 91%	600 °C		[13]
Rh/CeO_2–ZrO_2	97% 96%	750 °C		[14]
Pt/CeO_2	98% -	800 °C		[15]
Pt@Silicalite-1	55% 61%	700 °C		[16]
Rh-Ni/Al_2O_3	90% 80%	750 °C		[17]
Ru-Ni/CeO_2	~70% 80%	700 °C		[18]
Single-atom Rh/TiO_2	80% 98%	650°C		[19]
**AD Mo/Ni alloy/SBA15**	**~95% 97.7%**	**800 °C**	**Air as oxidants**	**This Work**

[a]CH_4 conversion, [b]CO selectivity

Table S5 Performance comparison between AD Mo/Ni alloy/SBA15 and Ni/SBA15 at different temperature with GHSV of 15,000 mL_{CH4} g_{cat}^{-1} h^{-1}

Temperature	Initial CH_4 conversion (%)		Initial H_2 selectivity (%)		Initial CO selectivity (%)	
Sample	AD Mo/Ni alloy/SBA15	Ni/SBA 15	AD Mo/Ni alloy/SBA15	Ni/SBA 15	AD Mo/Ni alloy/SBA15	Ni/SBA 15
800 °C	93.9	95.9	93.2	91.0	96.3	92.5
750 °C	90.5	88.1	91.2	89.3	90.9	87.8
700 °C	80.9	74.4	88.4	85.2	84.1	77.7

Table S6 Performance comparison between AD Mo/Ni alloy/SBA15 and Ni/SBA15 at different temperature with GHSV of 24,000 $mL_{CH4}\ g_{cat}^{-1}\ h^{-1}$

Temperature	Initial CH_4 conversion (%)		Initial H_2 selectivity (%)		Initial CO selectivity (%)	
Sample	AD Mo/Ni alloy/SBA15	Ni/SBA 15	AD Mo/Ni alloy/SBA15	Ni/SBA 15	AD Mo/Ni alloy/SBA15	Ni/SBA 15
800 °C	92.5	85.5	95.3	87.8	92.1	88.7
750 °C	83.9	68.5	88.2	78.2	84.5	84.3
700 °C	68.3	52.7	80.5	63.5	71.8	59.7

Table S7 Performance comparison between AD Mo/Ni alloy/SBA15 and Ni/SBA15 at different temperature with GHSV of 48,000 $mL_{CH4}\ g_{cat}^{-1}\ h^{-1}$

Temperature	Initial CH_4 conversion (%)		Initial H_2 selectivity (%)		Initial CO selectivity (%)	
Sample	AD Mo/Ni alloy/SBA15	Ni/SBA15	AD Mo/Ni alloy/SBA15	Ni/SBA15	AD Mo/Ni alloy/SBA15	Ni/SBA15
800 °C	88.9	84.3	88.6	87.4	89.0	88.7
750 °C	82.0	72.7	85.8	82.1	84.8	80.8
700 °C	73.4	59.6	81.5	74.4	77.8	69.2

Supplementary References:

[1] A. Emamdoust, V. La Parola, G. Pantaleo, M. L. Testa, S. Farjami Shayesteh, A. M. Venezia, *Journal of Energy Chemistry* **2020**, *47*, 1-9.

[2] V. La Parola, G. Pantaleo, A. M. Venezia, *Catalysts* **2018**, *8*, 220.

[3] R. K. Singha, A. Shukla, A. Yadav, L. N. Sivakumar Konathala, R. Bal, *Applied Catalysis B: Environmental* **2017**, *202*, 473-488.

[4] G. Pantaleo, V. L. Parola, F. Deganello, R. K. Singha, R. Bal, A. M. Venezia, *Applied Catalysis B: Environmental* **2016**, *189*, 233-241.

[5] L. Li, S. He, Y. Song, J. Zhao, W. Ji, C.-T. Au, *Journal of Catalysis* **2012**, *288*, 54-64.

[6] L. Li, Y. Yao, B. Sun, Z. Fei, H. Xia, J. Zhao, W. Ji, C.-T. Au, *ChemCatChem* **2013**, *5*, 3781-3787.

[7] A. Larimi, S. Alavi, *Int. J. Chem. Eng. Appl* **2012**, *3*, 6-9.

[8] H. Özdemir, M. A. F. Öksüzömer, M. A. Gürkaynak, *Fuel* **2014**, *116*, 63-70.

[9] G. Pantaleo, V. La Parola, F. Deganello, P. Calatozzo, R. Bal, A. M. Venezia, *Applied Catalysis B: Environmental* **2015**, *164*, 135-143.

[10] V. A. Kirillov, Z. A. Fedorova, M. M. Danilova, V. I. Zaikovskii, N. A. Kuzin, V. A. Kuzmin, T. A. Krieger, V. D. Mescheryakov, *Applied Catalysis A: General* **2011**, *401*, 170-175.

[11] Z. Wang, Y. Cheng, X. Shao, J.-P. Veder, X. Hu, Y. Ma, J. Wang, K. Xie, D. Dong, S. Ping Jiang, G. Parkinson, C. Buckley, C.-Z. Li, *Applied Catalysis A: General* **2018**, *565*, 119-126.

[12] S. Somacescu, N. Cioatera, P. Osiceanu, J. M. Calderon-Moreno, C. Ghica, F. Neaţu, M. Florea, *Applied Catalysis B: Environmental* **2019**, *241*, 393-406.

[13] Y. Hou, S. Ogasawara, A. Fukuoka, H. Kobayashi, *Catalysis Science & Technology* **2017**, *7*, 6132-6139.

[14] S. Eriksson, S. Rojas, M. Boutonnet, J. L. G. Fierro, *Applied Catalysis A: General* **2007**, *326*, 8-16.

[15] R. K. Singha, A. Shukla, A. Yadav, T. Sasaki, A. Sandupatla, G. Deo, R. Bal, *Catalysis Science & Technology* **2017**, *7*, 4720-4735.

[16] L. Ma, C. Ding, J. Wang, Y. Li, Y. Xue, J. Guo, K. Zhang, P. Liu, X. Gao, *International Journal of Hydrogen Energy* **2019**, *44*, 21847-21857.

[17] C. Alvarez-Galvan, M. Melian, L. Ruiz-Matas, J. L. Eslava, R. M. Navarro, M. Ahmadi, B. Roldan Cuenya, J. L. G. Fierro, *Front Chem* **2019**, *7*.

[18] G. Pauletto, N. Libretto, D. C. Boffito, J. T. Miller, A. Jentys, G. S. Patience, J. A. Lercher, *Applied Catalysis B: Environmental* **2021**, *286*, 119849.

[19] Y. Tang, V. Fung, X. Zhang, Y. Li, L. Nguyen, T. Sakata, K. Higashi, D.-e. Jiang, F. F. Tao, *Journal of the American Chemical Society* **2021**, *143*, 16566–16579.
